# Ultrasound-guided stellate ganglion acupuncture for chronic insomnia disorder using fNIRS: study protocol for a randomized controlled trial

**DOI:** 10.3389/fneur.2026.1855633

**Published:** 2026-06-29

**Authors:** Jiaqi Zhang, Jiacheng Song, Jun Hu, Wentao Zhang, Yongjie Feng, Wanqi Ding, Caixia Xu, Hao Liu

**Affiliations:** 1 Tongde Hospital of Zhejiang Province Affiliated to Zhejiang Chinese Medical University (College of Integrated Traditional Chinese and Western Medicine Clinical Medicine), Hangzhou, China; 2 Department of Acupuncture, Hangzhou Gongshu Hospital of Integrated Traditional and Western Medicine, Hangzhou, China; 3 Department of Acupuncture, Tongde Hospital of Zhejiang Province Affiliated to Zhejiang Chinese Medical University, Hangzhou, China

**Keywords:** acupuncture, brain function, functional near-infrared spectroscopy, insomnia, randomized controlled trial, stellate ganglion, ultrasound

## Abstract

**Background:**

Chronic insomnia disorder (CID) is common and often impairs daytime functioning and quality of life. Although cognitive behavioral therapy for insomnia and hypnotic medication are widely used, their clinical use may be limited by accessibility, cost, adherence, and concerns about long-term adverse effects. Acupuncture is commonly used for CID, but conventional acupuncture alone may require a relatively long treatment course. Because sympathetic hyperarousal plays an important role in insomnia, the stellate ganglion (SG) may represent a useful therapeutic target. This trial aims to evaluate the clinical efficacy and potential neurofunctional mechanisms of ultrasound-guided SG acupuncture combined with conventional acupuncture in patients with CID.

**Methods/design:**

This single-center, parallel-group randomized controlled trial will enroll 80 patients with CID, who will be allocated in a 1:1 ratio to a treatment group or a control group. In addition, healthy controls will be recruited for baseline functional near-infrared spectroscopy (fNIRS) comparison only. Before fNIRS acquisition, healthy controls will undergo clinical screening and baseline assessments. The control group will receive conventional acupuncture, whereas the treatment group will receive ultrasound-guided SG acupuncture plus conventional acupuncture. Both groups will receive 12 treatment sessions over 4 weeks. Outcomes will be assessed at baseline, week 4, and week 8. The primary outcomes are the Insomnia Severity Index (ISI) and fNIRS data of brain function. Secondary outcomes include the Pittsburgh Sleep Quality Index (PSQI), Hamilton Anxiety Rating Scale (HAMA), and overall clinical efficacy.

**Discussion:**

This trial will determine whether ultrasound-guided SG acupuncture combined with conventional acupuncture provides additional benefit over conventional acupuncture alone for CID. It will also explore treatment-related changes in brain function by combining clinical scales with fNIRS assessment. The findings may provide more objective evidence for acupuncture-based treatment of CID and help clarify its neurofunctional basis.

**Clinical trial registration:**

https://itmctr.ccebtcm.org.cn/mgt/dashboard, ITMCTR2025000535.

## 1 Introduction

Insomnia is a common sleep disorder characterized by difficulty initiating sleep, difficulty maintaining sleep, or early morning awakening, leading to poor sleep quality and impaired daytime functioning (1). When these symptoms occur at least three nights per week for 3 months or longer, the condition is classified as chronic insomnia disorder (CID) (2). According to World Health Organization estimates, CID affects approximately 10–15% of adults worldwide and is associated with substantial adverse outcomes, including cognitive impairment, mood disturbances and reduction in quality of life (3, 4).

Current treatments for insomnia include cognitive behavioral therapy for insomnia (CBT-I), pharmacotherapy, and traditional Chinese medicine approaches (5, 6). Although CBT-I is recommended as first-line therapy, its use is often limited by availability, time burden, and cost, which may reduce uptake and adherence (7–9). Medications can improve sleep in the short term, but long-term use is restricted by concerns about adverse effects, dependence, and cognitive impairment (10).

Acupuncture is widely used for CID because it is relatively low-cost and generally safe, and available evidence suggests that it can improve sleep quality and related symptoms (11–14). However, in view of the multifactorial etiology of CID, conventional acupuncture alone may require a relatively long treatment course, which may in turn affect adherence (15, 16).

Insomnia is frequently associated with sympathetic nervous system (SNS) hyperarousal (17). The stellate ganglion (SG), as a principal node of the SNS, has been implicated in autonomic regulation relevant to sleep (18). Stellate ganglion block (SGB) has been reported to reduce sympathetic outflow, improve sleep quality, shorten sleep latency, and relieve daytime dysfunction (19). However, its wider use is limited by procedure-related adverse effects such as hoarseness, dizziness, and puncture-site hematoma (20). In our clinical practice, SG acupuncture appears to facilitate sleep onset and improve sleep quality, and similar findings have been described in case-based clinical reports (21). Ultrasound-guided SG acupuncture may therefore offer a safer and more practical approach to autonomic modulation while complementing conventional acupuncture.

Most acupuncture trials in CID rely mainly on subjective scales. Functional near-infrared spectroscopy (fNIRS) offers a noninvasive way to assess cortical hemodynamics and functional connectivity (22). Insomnia has been associated with functional alterations in the prefrontal cortex (PFC) (23, 24), and task-evoked PFC activation and resting-state PFC connectivity may provide complementary neurofunctional information (25, 26). Accordingly, fNIRS combined with a verbal fluency task (VFT) has been used to detect reduced PFC activation in CID (25, 27). The VFT is feasible and engages executive-linguistic processes such as lexical retrieval, self-monitoring, and response inhibition (25, 27–29), making it a suitable paradigm for probing prefrontal function in this population.

Building upon these considerations, the present trial is designed to characterize the brain functional features of CID using fNIRS and to determine whether adding ultrasound-guided SG acupuncture to conventional acupuncture provides greater clinical benefit than conventional acupuncture alone. The study is also expected to generate objective neurofunctional evidence for this integrated treatment strategy.

## 2 Methods and analysis

### 2.1 Study design

The present study is designed as a single-center, parallel-group, randomized controlled trial to be conducted at Tongde Hospital of Zhejiang Province Affiliated to Zhejiang Chinese Medical University, during the period from June 1, 2025 to August 1, 2026. Ethical approval for the study protocol has been granted by the hospital Ethics Committee (No. 2024-098K), and trial registration has been completed in the International Traditional Medicine Clinical Trial Registry (ITMCTR2025000535). The study protocol was developed in accordance with the Standard Protocol Items: Recommendations for Interventional Trials (SPIRIT) guidelines and was guided by the principles of the Consolidated Standards of Reporting Trials (CONSORT) (30) and the Standards for Reporting Interventions in Clinical Trials of Acupuncture (STRICTA) (31).

### 2.2 Ethics approval

**Figure 1:**
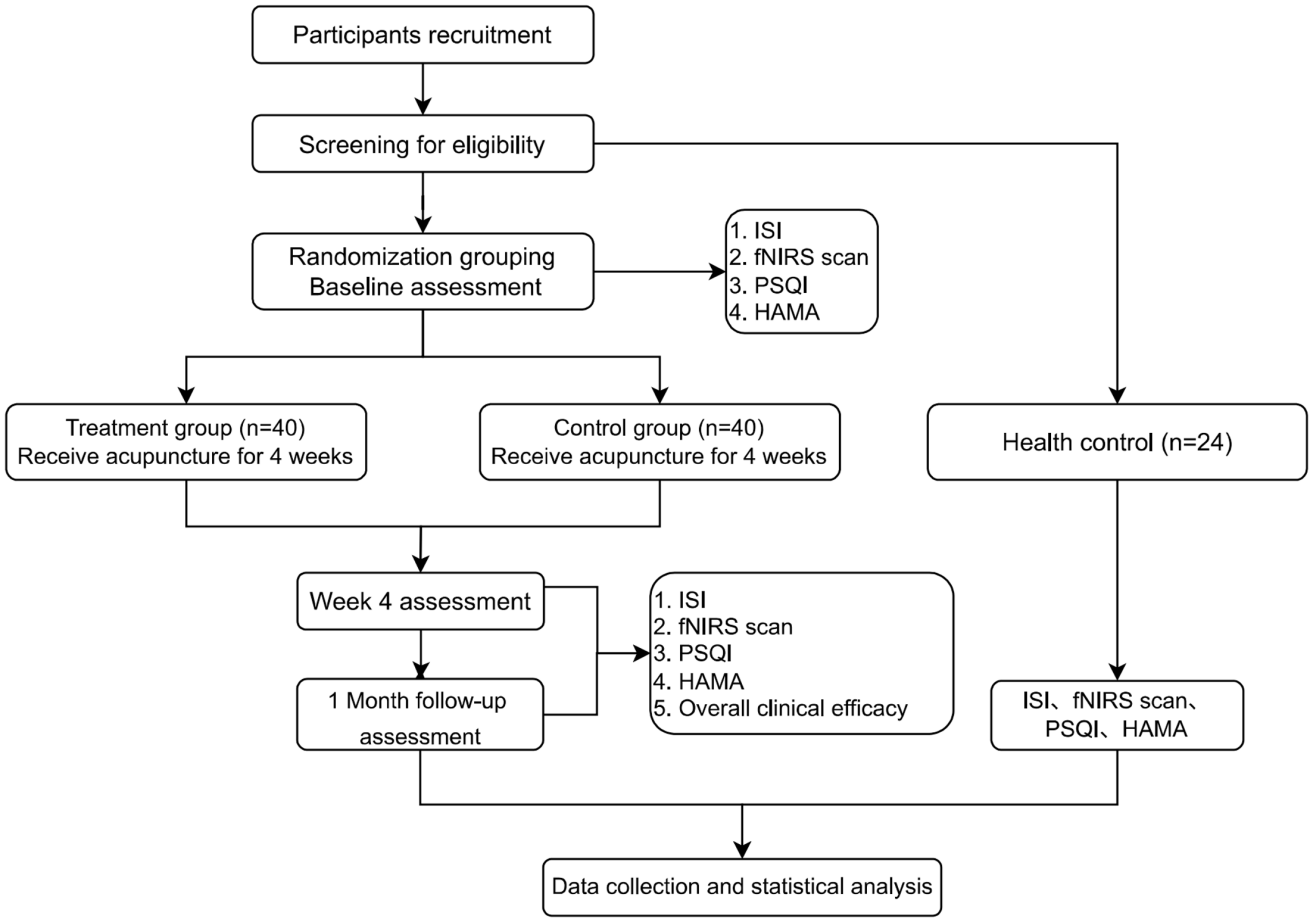
The flowchart of trial procedures.

Written informed consent will be obtained before enrollment. Patients with CID will be randomized to the treatment or control group. A healthy control cohort will undergo baseline clinical screening and fNIRS assessment only, without randomization, acupuncture treatment, or follow-up efficacy assessment. Study-related interventions, assessments, and consultations will be provided free of charge. The study flow is shown in Figure 1, and the assessment schedule is presented in Table 1.

**Table 1:** Schedule of enrolment, interventions and outcome assessments.

Timepoint	Baseline		Treatment phase	Follow-up phase
	Week −1	Week 0	Week 1–4	Week 8
Participants				
Eligibility screen	×			
Signed informed consent		×		
Clinical interview		×		
Physical examination		×		
Randomization		×		
Interventions				
treatment group		×	×	
Control group		×	×	
Primary outcomes				
ISI		×	×	×
fNIRS assessment		×	×	×
Secondary outcomes				
PSQI		×	×	×
HAMA		×	×	×
Overall clinical efficacy			×	×
Assessments				
Adverse events		×	×	
Use of sleep medications		×	×	×
Patients’ compliance		×	×	×

ISI, the insomnia severity index; PSQI, Pittsburgh sleep quality index; HAMA, Hamilton anxiety scale. × = Implementation of the corresponding project at the corresponding point in time.

### 2.3 Sample size calculation

This trial has two prespecified primary endpoints: change in Insomnia Severity Index (ISI) score and change in task-evoked prefrontal oxygenated hemoglobin (Oxy-Hb) response measured by fNIRS. A Bonferroni-style alpha-allocation strategy will be used to control the family-wise type I error rate, with a two-sided alpha of 0.025 assigned to each primary endpoint. For the ISI endpoint, a 3-point between-group difference was considered clinically meaningful, consistent with the lower bound of minimal clinically important difference estimates for the ISI (32), and supported by a randomized acupuncture trial in primary insomnia (33). Using a pooled SD of 3.8 from a comparable insomnia population (33), the standardized effect size was estimated as Cohen’s *d* = 3.0/3.8 = 0.79. With a two-sided *α* of 0.025, 1:1 allocation, and 80% power, approximately 32 participants per group are required.

For the fNIRS endpoint, a standardized effect size of *d* = 0.80 was assumed for the between-group difference in prefrontal Oxy-Hb change. With alpha = 0.025, 1:1 allocation, and 80% power, approximately 27 participants per group are required. Because the ISI endpoint requires the larger analyzable sample, it is the binding constraint. After allowing for a 20% attrition rate, the required enrollment sample size was calculated as follows:


$$n_{\text{enrolled}}=\frac{n_{\text{required}}}{1-\text{attrition rate}}=\frac{32}{1-0.20}=40$$

Thus, 40 participants per group, for a total of 80 participants, will be randomized.

Sensitivity analysis showed that, with 32 analyzable participants per group, the study would retain approximately 80.4% power for ISI and 81.4% power for fNIRS Oxy-Hb at alpha = 0.025. If the true fNIRS effect decreases to *d* = 0.75, power would decrease to approximately 75.7%, indicating that the fNIRS endpoint is more sensitive to effect-size assumptions. Strict fNIRS acquisition, preprocessing, and quality-control procedures will therefore be implemented. Previous fNIRS acupuncture studies have generally included 12–20 participants (34–36). On this basis, at least 20 participants per group will be included in the fNIRS analysis. After allowing for an anticipated dropout rate of 20%, 24 healthy controls per group will be planned for fNIRS assessment.

### 2.4 Participants

Patients with CID and healthy controls will be recruited through outpatient clinics and community channels, including WeChat and university campuses. Recruitment materials will describe the target population, eligibility criteria, intervention procedures, and contact details. Interested individuals will receive a study brochure and complete screening. Eligible patients will undergo baseline assessment and randomization, followed by assessments at baseline, week 4, and week 8. Healthy controls will undergo baseline clinical screening and baseline assessments before fNIRS acquisition.

### 2.5 Recruitment and procedure

Recruitment will take place at Tongde Hospital of Zhejiang Province Affiliated to Zhejiang Chinese Medical University through outpatient clinics and online notices distributed via WeChat. Recruitment materials will describe the target population, eligibility criteria, intervention procedures, and contact details. Interested individuals will receive a study brochure and complete a screening form. Eligible patients with CID will then undergo baseline assessment and randomization. Outcome assessments will be conducted at baseline, week 4 (post-treatment), and week 8 (follow-up). Healthy controls will be recruited as a reference group for baseline fNIRS comparison only. Before fNIRS assessment, their eligibility will be verified through clinical interview and baseline assessments.

### 2.6 Eligibility

#### 2.6.1 Diagnostic criteria

CID will be diagnosed in accordance with the International Classification of Sleep Disorders, Third Edition (ICSD-3). Specifically, eligibility will require fulfillment of all of the following criteria:

1. Impairment in sleep initiation, sleep maintenance, and/or early-morning awakening.
2. Symptom occurrence on at least three nights per week for a duration of ≥3 months.
3. Absence of attribution of the insomnia-related complaints to other sleep disorders.

#### 2.6.2 Eligibility criteria

Participants will be considered eligible for inclusion upon fulfillment of all of the following criteria:

1. Diagnosis of CID in accordance with the ICSD-3 diagnostic criteria, together with an ISI score of ≥10.
2. Age between 18 and 70 years, with no sex-based restriction, and right-handedness.
3. No use of medications or other therapeutic interventions for insomnia within 1 month prior to enrollment.
4. Preserved ability for normal communication and compliance with study procedures.
5. Written informed consent must be provided.

Healthy controls will be required to meet all of the following criteria:

1. Right-handed individuals of either sex, aged between 18 and 70 years;
2. An ISI score ≤7, a Pittsburgh Sleep Quality Index (PSQI) score ≤5, and a HAMA score <7;
3. No history of major chronic diseases or severe organic dysfunctions (e.g., cardiovascular, hepatic, or renal diseases);
4. No use of hypnotics, antidepressants, central nervous system stimulants, or any other medications/substances known to substantially affect sleep architecture or central nervous system function within 1 month prior to enrollment;
5. Voluntary provision of written informed consent prior to study participation.

#### 2.6.3 Exclusion criteria

Participants will be excluded upon fulfillment of any of the following criteria:

1. Presence of severe or unstable medical conditions, including cardiovascular or cerebrovascular disease, malignancy, nephritis, hematological disorders, other life-threatening diseases, or active infectious disease.
2. Presence of severe depressive symptoms, defined as a Hamilton Depression Rating Scale (HAMD) score of >24.
3. Presence of significant impairment in speech, language, or hearing, or the presence of cognitive or neurological disorders.
4. Current substance abuse or severe dependence on alcohol or other psychoactive substances, or current exposure to medications or substances with substantial effects on sleep or central nervous system function, including antidepressants or central stimulants.
5. Presence of secondary insomnia attributable to other organic diseases.
6. Pregnancy or breastfeeding status.
7. History of needle syncope or presence of skin damage at the intended acupuncture sites.
8. Known allergy to metals, including acupuncture needles.
9. Current participation in another clinical study.

#### 2.6.4 Termination criteria

Trial termination may be considered under any of the following circumstances:

1. Occurrence of serious safety concerns.
2. Evidence of futility of trial continuation due to insufficient clinical benefit.
3. Occurrence of major protocol errors or substantial deviations in trial conduct that compromise scientific validity.
4. Discontinuation requested by the sponsor or investigator for operational reasons, including funding or management constraints.
5. Suspension or termination mandated by the relevant regulatory or administrative authority.

### 2.7 Randomization and blinding

Eligible patients with CID will be randomly assigned 1:1 to the treatment or control group. An independent statistician not involved in recruitment, intervention, outcome assessment, or data analysis will generate the randomization sequence. Allocation will be concealed in sequentially numbered, opaque, sealed envelopes. After baseline assessment, a research assistant not involved in outcome assessment or data analysis will open the next envelope and assign the participant.

Treatments will be delivered in separate sessions to reduce contamination. Acupuncturists cannot be blinded because the procedures differ, but participants will not be informed of the study hypothesis or expected superiority of either intervention. Outcome assessors and statisticians will remain blinded throughout the trial. Accidental unblinding will be documented.

### 2.8 Intervention

**Figure 2:**
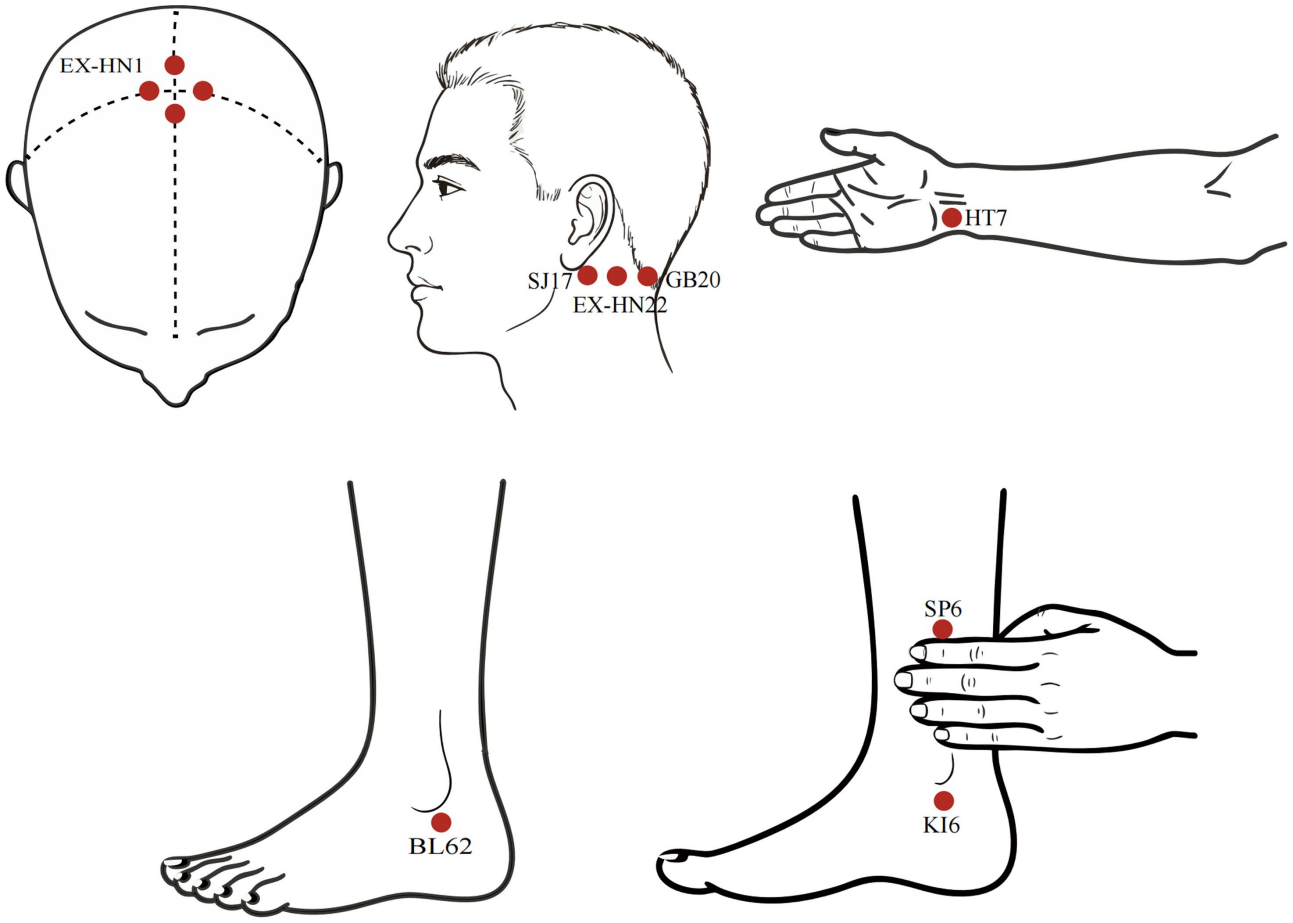
Locations of selected acupoints.

Licensed acupuncturists with at least 5 years of clinical experience will receive standardized training before trial initiation. Both groups will receive the same conventional acupuncture prescription: Sishencong (EX-HN1), Anmian (EX-HN22, bilateral), Shenmen (HT7, bilateral), Sanyinjiao (SP6, bilateral), Shenmai (BL62, bilateral), and Zhaohai (KI6, bilateral). Detailed acupoint locations are shown in Table 2 and Figure 2. Treatment will include 12 sessions over 4 weeks, three sessions per week. In the treatment group, ultrasound-guided SG acupuncture will be performed first, followed immediately by conventional acupuncture; after all needles have been inserted, needles will be retained concurrently for 30 min.

**Table 2:** Locations of selected acupoints.

Acupoint	Location
Sishencong (EX-HN1)	Located on the head, at four points situated 1 cun anterior, posterior, and lateral to Baihui (GV20).
Anmian (EX-HN22)	At the halfway point between Fengchi (GB20) and Yifeng (SJ17)
Shenmen (HT7)	Located on the anterior wrist region, at the ulnar end of the transverse crease of the palm, on the radial side of the tendon of the flexor carpi ulnaris.
Shenmai (BL62)	Located in the ankle region, directly inferior to the prominence of the lateral malleolus, in the depression between the lower border of the lateral malleolus and the calcaneus.
Zhaohai (KI6)	Located in the ankle region, inferior to the prominence of the medial malleolus, in the depression along the lower border of the medial malleolus.
Sanyinjiao (SP6)	Located on the medial aspect of the lower leg, 3 cun superior to the prominence of the medial malleolus, posterior to the medial border of the tibia.

cun: proportional bone (skeletal) cun. Baihui (GV20): located on the head, 5 cun directly superior to the midpoint of the anterior hairline. Fengchi (GB20): located in the posterior cervical region, inferior to the occipital bone, in the depression between the upper portion of the sternocleidomastoid muscle and the upper portion of the trapezius muscle. Yifeng (SJ17): located in the neck region, posterior to the earlobe, in the depression anterior to the inferior aspect of the mastoid process.

#### 2.8.1 Control group

Participants in the control group will receive standardized conventional acupuncture. Sterile single-use needles (0.25 × 25 mm, Jiachen Acupuncture Instruments Co., Ltd., China) will be used at EX-HN1, EX-HN22, HT7, BL62, and KI6, whereas 0.25 × 40 mm (Jiachen Acupuncture Instruments Co., Ltd., China) filiform needles will be used at SP6. Needling depth and angle will be adjusted within a prespecified range according to the anatomical features of each point and the participant’s local tissue thickness. Specifically, EX-HN1 will be inserted obliquely at an angle of 15°–30° along the subgaleal plane, with a target depth of 15–20 mm. At EX-HN22, the needle will be inserted perpendicularly or slightly obliquely to a depth of 10–15 mm, taking care to avoid excessively deep anterior needling. HT7 will be needled perpendicularly to 8–12 mm. BL62 and KI6 will also be inserted perpendicularly, generally to a depth of 8–12 mm. For SP6, perpendicular insertion will be performed to a depth of 25–30 mm. For bilateral acupoints, the right side will be needled first, followed by the left side. After deqi is obtained, needles will be retained for 30 min and removed, with brief compression using sterile gauze when needed.

Deqi will be elicited gently by lifting-thrusting and twirling manipulation after the intended depth is reached. Lifting-thrusting will use an amplitude of about 2–3 mm, and twirling will be kept within 90–180 degrees at approximately 1 Hz for 10 s. Manual stimulation will be applied after insertion and repeated at 10 and 20 min, giving each acupoint about 30 s of planned stimulation per session.

Deqi refers to the characteristic needling response induced by appropriate needle manipulation, including lifting-thrusting and rotation, after achievement of the intended insertion depth (37). Immediately after each manipulation, participants will rate typical needling sensations, including soreness, numbness, distension, heaviness, warmth, and radiating sensation, on a 0–10 numerical rating scale. At the same time, the acupuncturist will evaluate needle-grasp sensation, defined as the pulling, heaviness, tightness, or increased resistance perceived under the needle during manipulation, using a 0–3 scale. For the purpose of procedural standardization, Deqi will be regarded as achieved when at least one typical patient-reported sensation is rated ≥3 and/or the operator-rated needle-grasp score is ≥1 (38, 39).

#### 2.8.2 Treatment group

**Figure 3:**
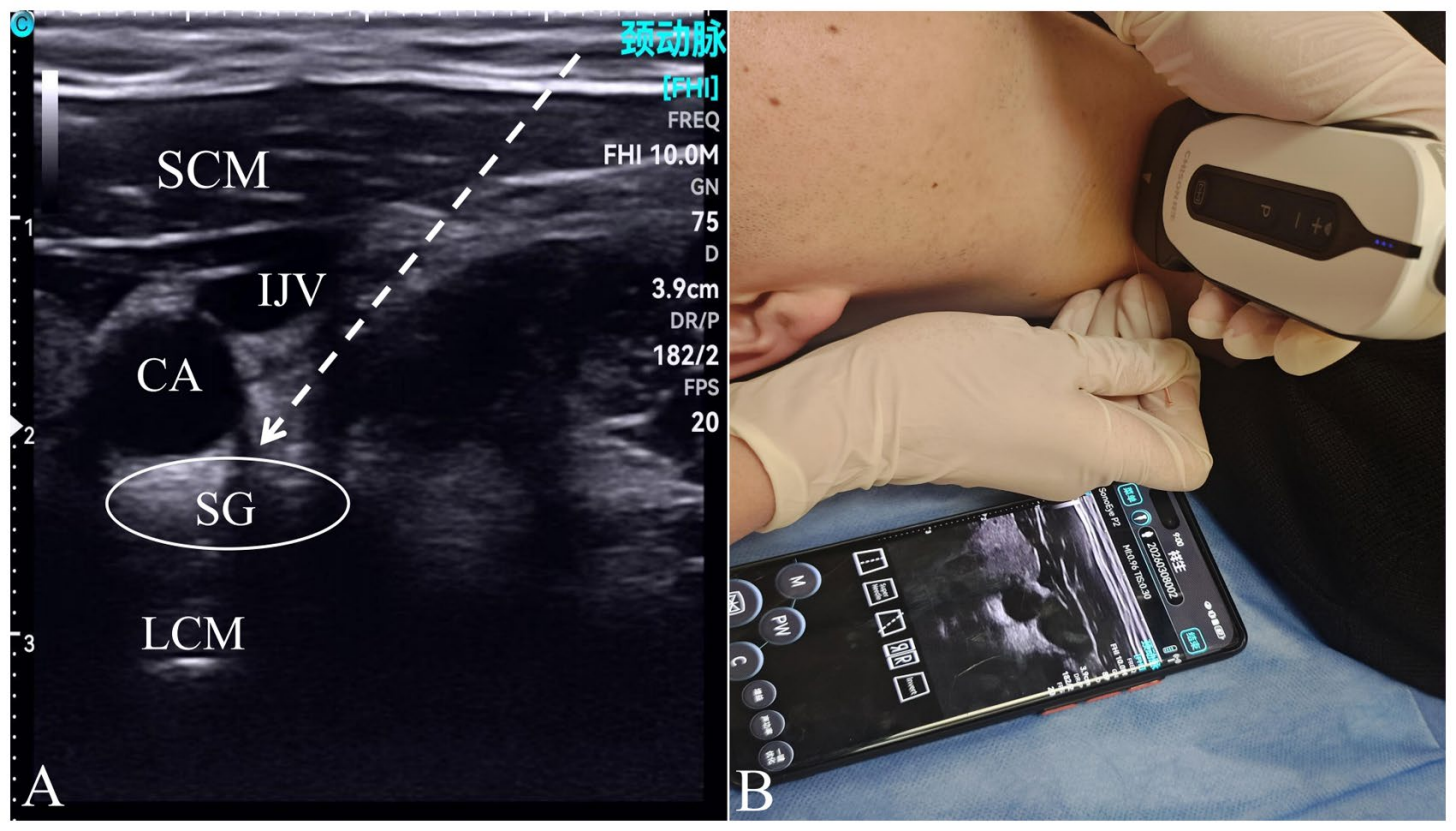
Ultrasound-guided SG acupuncture. **(A)** Sonographic illustration of SG; **(B)** Ultrasound-guided acupuncture at the SG. SG, stellate ganglion; SCM, sternocleidomastoid muscle; IJV, internal jugular vein; CA, carotid artery; LCM, longus colli muscle.

In addition to conventional acupuncture, the treatment group will receive ultrasound-guided SG acupuncture. The pathway was adapted from ultrasound-guided SG block techniques (40–42). Participants will lie supine with the neck slightly extended. A SonoEye V2 musculoskeletal ultrasound system (Wuxi Xiangsheng Medical Technology Co., Ltd., China) with a high-frequency linear probe and sterile single-use needles (0.25 × 40 mm; Jiachen Acupuncture Instruments Co., Ltd., Wujiang, China) will be used. After skin disinfection and sterile probe preparation, the probe will be placed transversely at the C6 level to visualize the carotid artery, internal jugular vein, thyroid gland, trachea, longus colli muscle, and prevertebral fascia. Under real-time in-plane ultrasound guidance, the needle will be advanced along a confirmed safe trajectory toward the prevertebral fascial plane adjacent to the longus colli muscle, while avoiding the carotid sheath, thyroid gland, and tracheoesophageal groove. Needling angle and depth will be individualized by real-time visualization. Gentle manipulation will be limited to 1–2 mm lifting-thrusting, within 90 degrees of rotation, at about 1 Hz for 10 s. Ultrasound-observed soft-tissue micro-movement during manipulation will be recorded as a procedural quality indicator, consistent with evidence that needle-tissue engagement can be quantified (43). The right side will be treated first, followed by the left side (Figures 3A,B); needles will then be retained bilaterally for 30 min without further manipulation.

Before each session, a safety checklist will identify bleeding tendency, anticoagulant use, recent neck trauma, cardiopulmonary instability, or other risk factors. Participants will be instructed to avoid swallowing, coughing, or neck movement during stepwise needle advancement. The procedure will stop immediately if severe pain, electric shock-like sensation, respiratory distress, or loss of continuous needle-tip visualization occurs. Local pressure will be applied after needle removal.

#### 2.8.3 Functional near-infrared spectroscopy

**Figure 4:**
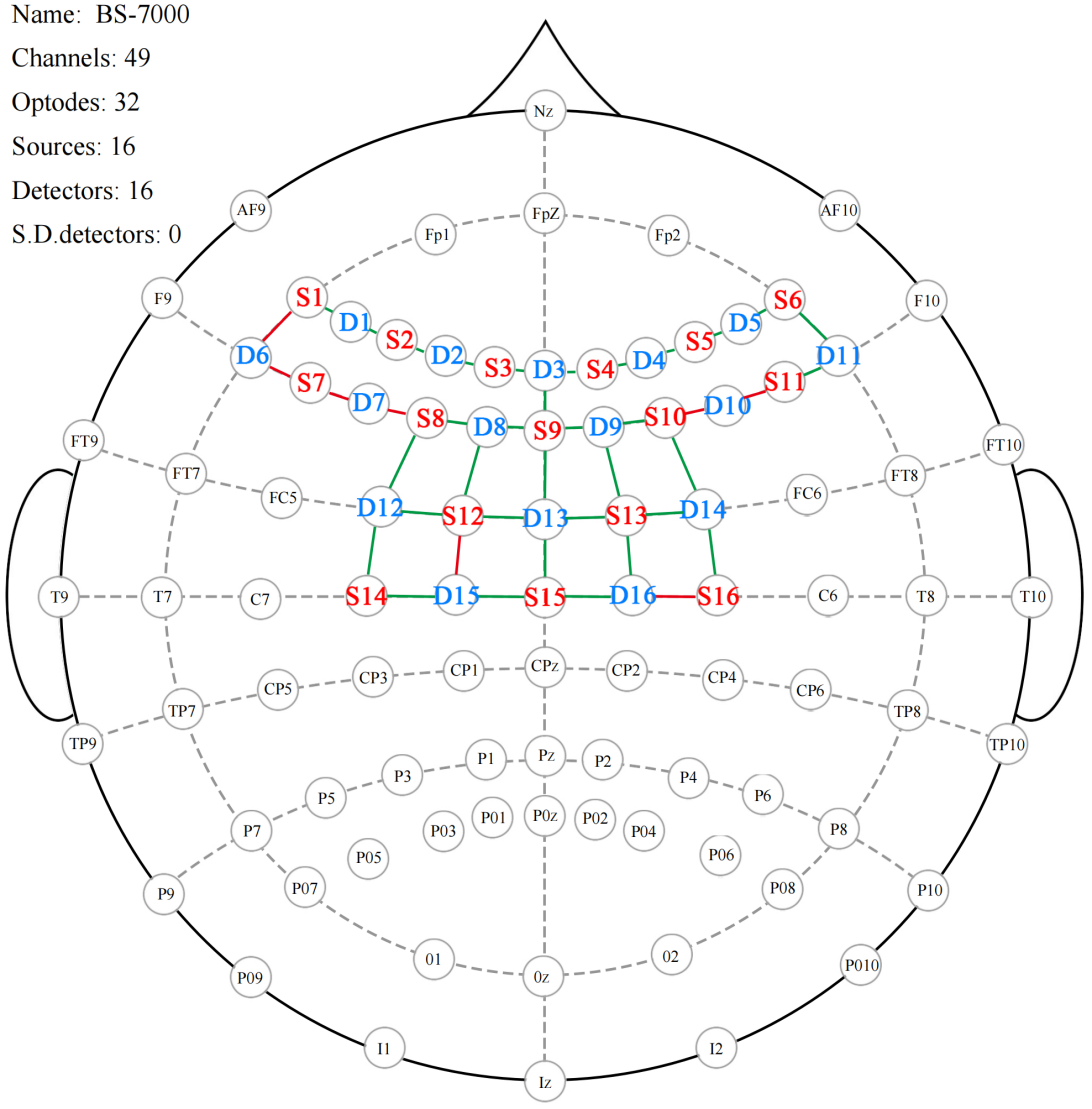
The positions of the emitters, detectors, and the 49 channels.

fNIRS data will be collected using the BS-7000 system (Wuhan Znion Technology Co., Ltd., Wuhan, China). Healthy controls will undergo baseline fNIRS assessment only, whereas patients with CID will be assessed at baseline, week 4, and week 8. Participants will avoid caffeine for at least 12 h, alcohol for at least 24 h, and strenuous exercise for 1 day before assessment. Recording time, caffeine/alcohol intake, medication use, and overnight sleep duration will be documented. Repeated assessments will be scheduled at similar times of day when feasible (Figure 4).

After a 5-min seated rest, a flexible cap with 16 sources and 16 detectors will be positioned according to the international 10–20 system, with detector 3 aligned to Fpz. The optode layout will cover frontal, temporal, and parietal cortices, with long-separation source-detector distance of approximately 30 mm. Dual wavelengths of 690 and 830 nm will be used, with manufacturer-preset sampling at 10 Hz. Cap repeatability will be controlled by using the same cap size, recording landmarks and optode positions, taking standardized photographs, and using the same trained operator when possible. Raw light intensity, optode-scalp coupling, and signal-to-noise ratio will be checked before recording.

**Figure 5:**
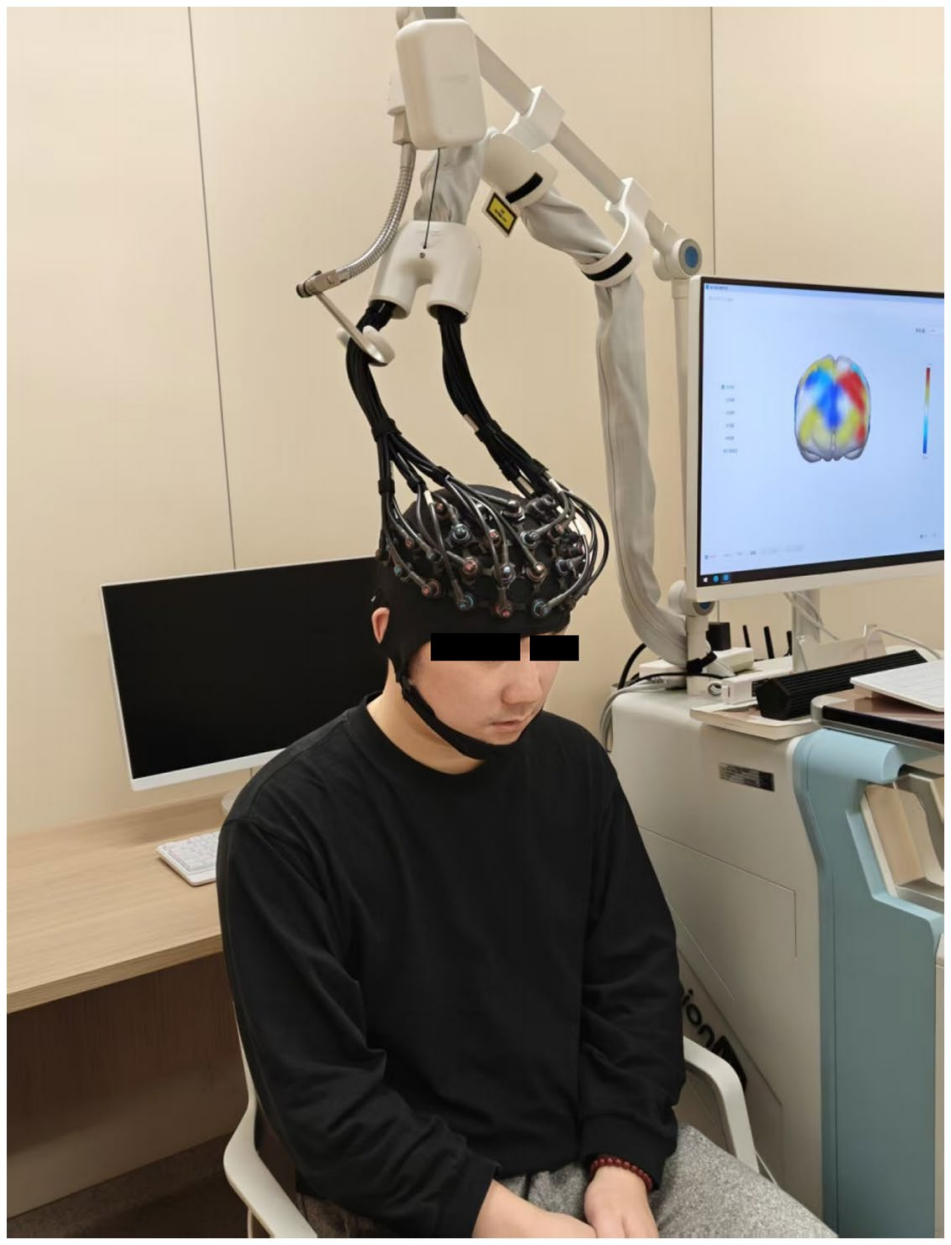
Shows a subject being tested by fNIRS.

**Figure 6:**
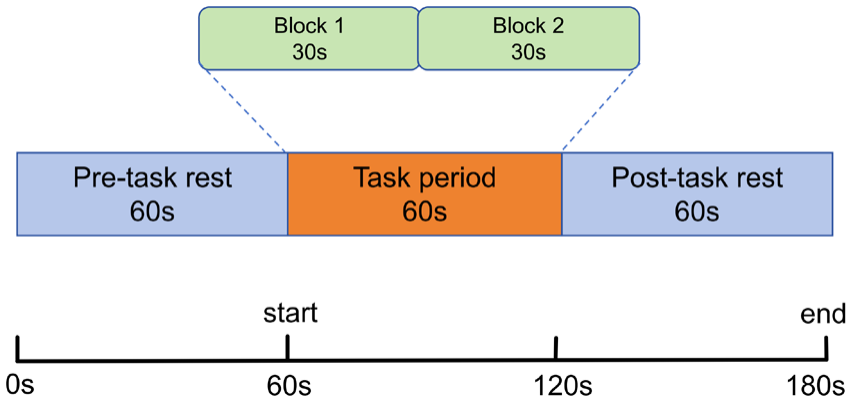
Flowchart of the resting-state test and verbal fluency task.

The protocol will include resting-state recording and a VFT (Figure 5). Before each recording, raw light intensity and optode-scalp coupling will be verified before each recording; channels will be retained only if light intensity falls within the manufacturer-recommended dynamic range and SNR ≥ 30 dB. A 60-s pre-task control period will be conducted under eyes-open conditions, during which participants softly repeat “1-2-3-4-5” to control for speech-related motor activity. The task will consist of two consecutive 30-s blocks, in which participants generate as many words as possible from predefined semantic categories. A 60-s post-task period will follow using the same control articulation. Six familiarity-matched categories (food, animals, household items, occupations, fruits/vegetables, and sports/exercise) will be counterbalanced using a computer-generated Latin-square schedule, so that each patient completes all six categories once across the three assessments. Task onset and duration will be recorded for individual-level generalized linear model (GLM) analysis (Figure 6).

### 2.9 Outcome measures

An independent, blinded clinician will perform all outcome assessments. Randomized CID patients will be evaluated at baseline, week 4, and week 8. Healthy controls will only undergo baseline screening and fNIRS, using the ISI, PSQI, and HAMA strictly to confirm eligibility and baseline characteristics. Additionally, self-reported sleep duration (extracted from the PSQI) will be recorded for all participants at baseline, and at weeks 4 and 8 for the randomized group.

#### 2.9.1 Primary outcomes

##### 2.9.1.1 ISI

The ISI is a validated 7-item scale assessing insomnia severity over the preceding 2 weeks. Items are scored from 0 to 4, yielding a total score of 0–28, with higher scores indicating greater severity. Scores are interpreted as no clinically significant insomnia (0–7), mild insomnia (8–14), moderate insomnia (15–21), and severe insomnia (22–28).

#### 2.9.2 fNIRS data of brain function

fNIRS will quantify task-evoked cortical hemodynamic responses, with Oxy-Hb as the primary hemodynamic indicator because of its sensitivity to task-related changes. These measures will characterize cortical activation and functional connectivity patterns in CID and explore neurofunctional changes after ultrasound-guided SG acupuncture plus conventional acupuncture compared with conventional acupuncture alone.

#### 2.9.3 Secondary outcomes

##### 2.9.3.1 PSQI

The Pittsburgh Sleep Quality Index (PSQI) is a self-report instrument for the evaluation of sleep quality during the preceding month. The instrument comprises 19 self-rated items, from which seven component scores are generated, each with a score range of 0 to 3. Thereafter, summation of the component scores results in a global score ranging from 0 to 21, with higher scores reflecting poorer sleep quality.

##### 2.9.3.2 HAMA

The Hamilton Anxiety Rating Scale (HAMA) is a clinician-rated instrument for the assessment of the severity of anxiety symptomatology. The scale consists of 14 items, each scored on a scale from 0 to 4, thereby yielding a total score ranging from 0 to 56. Total scores are generally categorized as follows: <17, mild anxiety; 18–24, mild-to-moderate anxiety; 25–30, moderate-to-severe anxiety; and ≥31, severe anxiety.

##### 2.9.3.3 Overall clinical efficacy

Overall clinical efficacy will be assessed at weeks 4 and 8 as an exploratory categorical outcome adapted from the Guidelines for Clinical Research of New Traditional Chinese Medicine Drugs (2020 Revision). To reduce baseline heterogeneity, sleep-duration improvement will be calculated from each participant’s own baseline value using the raw PSQI sleep-duration item. Outcome categories will be operationally defined as follows:

- *Ineffective*: No improvement in sleep disturbance or related symptoms, or the presence of symptom exacerbation.
- *Effective*: An increase in total sleep time of 1–3 h compared with baseline, with concomitant improvement in related symptoms.
- *Markedly effective*: An increase in total sleep time of 3–6 h, together with significant improvement in related symptoms.
- *Cured*: Restoration of sleep duration to a near-normal level (≥6 h), with near-complete resolution of related symptoms.

Accordingly, the overall effective rate will be derived from the proportion of participants categorized as Effective, Markedly effective, or Cured within the total randomized sample, followed by multiplication by 100% for percentage expression.

### 2.10 Assessment of safety

All adverse events (AEs) during intervention and follow-up will be recorded, including onset, duration, severity, management, outcome, and causality. AEs will be graded as mild, moderate, or severe. Mild AEs include transient symptoms requiring no specific treatment, such as local pain, minor ecchymosis, or transient dizziness. Moderate AEs include symptoms requiring clinical management or observation, such as persistent local pain, moderate hematoma, vasovagal reaction, transient hoarseness, dysphagia, or ipsilateral ocular symptoms. Severe AEs include marked functional impairment, hospitalization, or serious events such as suspected neurovascular injury, airway compromise, severe dyspnea, expanding cervical hematoma, pneumothorax, or loss of consciousness.

Common needle-related AEs, including minor bleeding and localized pain, will be managed according to standard clinical protocols. In cases of dizziness or needle syncope, the procedure will be immediately terminated; the participant will be placed in a supine position with continuous monitoring of vital signs and oxygen saturation (SpO2) until complete recovery. Supportive care will be provided as necessary, and participants with persistent symptoms will be referred to the emergency department for further evaluation. Specific attention will be given to AEs related to ultrasound-guided SG acupuncture, such as hoarseness, dysphagia, dyspnea, choking sensations, radiating paresthesia, and symptoms suggesting recurrent laryngeal nerve, phrenic nerve, vascular, or pleural involvement. Upon occurrence of these symptoms, needle manipulation will cease immediately, and the needle will be withdrawn under ultrasound guidance. Vital signs, airway patency, and neurological functions will be assessed immediately, with oxygen therapy administered if indicated. Participants exhibiting persistent complications (e.g., progressive neck swelling, suspected pneumothorax, or unstable vital signs) will be transferred to the emergency department and referred to appropriate specialists, including anesthesiology, otolaryngology, or vascular surgery.

Vital signs will be measured before and after each treatment, and participants receiving SG acupuncture will be observed for at least 30 min. Serious AEs will be reported to the ethics committee within 24 h. Participants who discontinue or suspend components for safety reasons will continue to receive appropriate medical evaluation and will be encouraged to complete outcome follow-up whenever ethically and clinically appropriate.

### 2.11 Data analysis

All statistical analyses will be performed by an independent statistician using SPSS version 29.0 (IBM Corp., Armonk, NY, USA) and R software (version 4.3.x). The primary analysis will follow the intention-to-treat principle, including all randomized participants; per-protocol analysis will be used as sensitivity analysis. Continuous variables will be summarized as mean ± SD or median (IQR), and categorical variables as counts and percentages. Baseline characteristics will be summarized descriptively, with standardized mean differences used to assess group balance. No formal baseline hypothesis testing will be performed. Baseline fNIRS comparisons between patients with CID and healthy controls will use ANCOVA controlling for age, sex, BMI, caffeine/alcohol intake, and recording time.

Longitudinal continuous outcomes will be analyzed using linear mixed-effects models with fixed effects for group, time, and group-by-time interaction, and a random intercept for each participant. The main treatment effect will be the between-group difference in change from baseline to week 4; week 8 will be analyzed as follow-up. An unstructured covariance matrix will be attempted first; if convergence fails, AR(1) or compound symmetry structures will be considered based on model fit. Missing data will be handled under the missing-at-random assumption using multiple imputation by chained equations with 20 datasets. The imputation model will include treatment group, time, baseline and available follow-up values, and relevant variables such as age, sex, ISI, PSQI, and HAMA. Results will be pooled using Rubin’s rules.

This is a superiority trial. For ISI, the null hypothesis is no between-group difference in change from baseline to week 4; the alternative is greater improvement in the treatment group. A 3-point ISI difference will be used for sample-size estimation and clinical interpretation but not as a noninferiority or superiority margin. Because there are two primary endpoints, a two-sided Bonferroni-adjusted alpha of 0.025 will be used for each. Secondary outcomes and exploratory fNIRS endpoints will be adjusted using the false discovery rate method where appropriate.

Raw fNIRS data will be processed using NirMaster software (Znion, Wuhan, China). To ensure transparency and reproducibility, the data analysis pipeline will follow standard fNIRS processing procedures, structured into preprocessing, quality control, and feature extraction.

Data Preprocessing: First, the raw light intensity data will be converted to optical density (OD). Second, motion artifacts will be identified and corrected using spline interpolation. Third, a band-pass filter (0.01–0.1 Hz) will be applied to the OD data to reduce physiological noise (e.g., heartbeat, respiration) and low-frequency signal drift. Finally, the filtered OD data will be converted to relative concentration changes in oxy-Hb, deoxyhemoglobin (deoxy-Hb), and total hemoglobin (tHb) using the modified Beer–Lambert law. The differential pathlength factor (DPF) will be set to 6.0 for both 690 nm and 830 nm wavelengths.

Quality Control: Channel signal quality will be evaluated using the coefficient of variation (CV), which will be calculated based on the raw light intensity during the initial resting period. Channels with a CV > 15% will be considered to have excessive noise and will be excluded from further analyses (44). Participants with more than 20% of their channels categorized as low-quality will be entirely removed from the group-level fNIRS analysis.

Feature Extraction: In accordance with standard practices, oxy-Hb will serve as the primary indicator of cortical activation due to its higher sensitivity to task-related changes (45). To quantify task-evoked activation during the VFT, a GLM will be applied at the individual level. The design matrix will include the task conditions convoluted with a canonical hemodynamic response function (cHRF). Task-related regression coefficients (*β* values) will be extracted to represent the activation strength for each channel. For region-of-interest (ROI) analyses, the extracted *β* values from channels located within the bilateral dorsolateral prefrontal cortex (DLPFC) and medial prefrontal cortex (mPFC) will be averaged.

Longitudinal comparisons of ROI metrics (baseline, week 4, and week 8) will be conducted using independent-samples *t*-tests or Mann–Whitney *U* tests for comparisons between groups at each time point, with FDR correction applied to multiple fNIRS comparisons.

### 2.12 Management of intervention and outcome changes

The trial will follow the approved protocol. Any change to intervention procedures, eligibility criteria, outcomes, or assessment schedule will require ethics committee approval. Safety-driven immediate changes will be implemented first and reported promptly. All amendments will be documented with rationale, details, and version updates, and the registry and statistical analysis plan will be updated before database lock and unblinding. Protocol deviations will be recorded and reported in the final publication.

## 3 Discussion

CID places a substantial burden on both individuals and society. It can markedly impair quality of life and work performance (46) and has been associated with a higher risk of metabolic syndrome (47), hypertension (48), and diabetes (49). Persistent untreated insomnia has also been linked to psychiatric morbidity, particularly depression (50), and may contribute to reduced pain tolerance and impaired immune function (51, 52). In addition, insomnia is often accompanied by anxiety, substance misuse, and other psychological or behavioral problems, including suicidal behavior in severe cases (53, 54).

The conventional acupuncture prescription in this trial includes EX-HN1, EX-HN22, HT7, SP6, BL62, and KI6. It was selected by integrating traditional Chinese medicine theory, clinical experience, and contemporary evidence. In traditional theory, insomnia is related to disturbance of the Heart and Brain and may involve dysfunction of the Liver, Spleen, and Kidney. EX-HN1 and EX-HN22 are commonly used for head-related and sleep-related symptoms; HT7 is used to calm the mind; SP6 regulates yin-yang balance; and BL62/KI6 correspond to the Yangqiao and Yinqiao meridians, which are traditionally linked to sleep–wake regulation.

Systematic reviews of pattern-based acupuncture prescriptions for insomnia have reported that HT7, SP6, EX-HN22, and EX-HN1 are among the commonly selected acupoints in clinical studies (55). A systematic review of randomized controlled trials on primary insomnia has indicated that BL62 and KI6 constitute a frequently used acupoint combination in clinical practice (56). These findings suggest that the present prescription is consistent with the core acupoint-selection patterns observed in previous insomnia studies. In addition, mechanistic studies and reviews indicate that acupuncture at sleep-related points, particularly combinations involving HT7 and SP6, may be associated with modulation of autonomic activity, sleep-related neurotransmitters, neuroendocrine function, and central arousal networks (57, 58).

The stellate ganglion is a major paravertebral sympathetic ganglion and is anatomically relevant to autonomic regulation. Evidence from SGB studies suggests that modulation of this region may influence sympathetic outflow and sleep-related symptoms (59), while experimental studies of electroacupuncture and autonomic neuromodulation indicate that stimulation parameters, including frequency and intensity, can affect sympathetic neural activity (60, 61). In addition, animal and clinical studies of acupuncture for insomnia have reported possible involvement of sleep-related neurochemical pathways, including reduced norepinephrine activity, enhanced GABAergic signaling, and regulation of melatonin secretion (58, 62). These findings provide a mechanistic rationale for incorporating SG acupuncture into CID management. Importantly, ultrasound guidance enables real-time visualization of surrounding anatomical structures and is expected to enhance procedural precision and safety during SG needling. However, because current evidence is indirect and this trial does not measure specific autonomic or neurochemical markers, the pathway linking ultrasound-guided SG acupuncture to sleep improvement remains a biologically plausible hypothesis rather than a confirmed mechanism.

To date, most clinical studies on insomnia have focused either on SG block or on conventional acupuncture alone. Evidence remains limited as to whether SG acupuncture combined with conventional acupuncture can further improve outcomes in CID. This trial therefore evaluates an integrated regimen of ultrasound-guided SG acupuncture plus conventional acupuncture, aiming to combine targeted autonomic modulation with broader symptom regulation. In addition to clinical rating scales, fNIRS will be used to characterize brain functional features, identify disease-related neuroimaging signatures, and compare changes before and after treatment. This multimodal design may improve the objectivity of outcome assessment and help clarify the neurofunctional correlates of acupuncture-related improvement in CID.

The present study is designed as a pragmatic add-on superiority trial in which both groups receive standardized conventional acupuncture, with the treatment group receiving additional ultrasound-guided SG acupuncture. Accordingly, any observed between-group difference reflects the add-on effect of SG acupuncture within this context, rather than the isolated effect of either modality. Several limitations warrant consideration. First, this is a single-center trial with a modest sample size, which may limit generalizability; furthermore, given the absence of published effect-size data for ultrasound-guided SG acupuncture in CID, the sample was powered to detect a clinically meaningful add-on difference in ISI, and smaller neurofunctional effects may be underpowered. Second, the absence of a sham SG acupuncture or para-needling control precludes determination of stellate-ganglion target specificity and prevents full separation of specific physiological effects from nonspecific needling, expectancy, therapist–participant interaction, or ultrasound-related contextual factors. Third, the short follow-up duration (final assessment at week 8) leaves the long-term durability of treatment effects uncertain. Fourth, fNIRS-derived Oxy-Hb signals reflect local cortical hemodynamics rather than direct neural activity, and are subject to confounding by systemic vascular status or CID-related neurovascular alterations; Oxy-Hb changes should therefore not be equated with neural activation or used to draw definitive causal inferences. Relatedly, the verbal fluency task engages prefrontal executive-linguistic networks specifically and may be influenced by semantic memory, linguistic proficiency, and educational background; task-evoked fNIRS responses should thus be interpreted as domain-specific rather than as a generalized index of executive function. Finally, as no universally accepted protocol for SG acupuncture currently exists, the manipulation parameters adopted here are protocol-defined, and optimal stimulation intensity, frequency, and treatment course remain undetermined.

Future research should employ multicenter, sham-controlled designs with larger samples, longer follow-up periods, and objective sleep measurements to confirm and extend these findings.

## References

[1] Zhang P Li Y Wu H. Guidelines for the diagnosis and treatment of insomnia in Chinese adults (2017th edition). Chin J Neurol. (2018) 51:324–35.

[2] Lee R Larson O Dhaliwal S Moon K Gerardy B de Chazal P . Comparative analysis of sleep physiology using qualitative and quantitative criteria for insomnia symptoms. Sleep. (2025) 48:1–10. doi: 10.1093/sleep/zsae301, 39713965 PMC11893537

[3] Carvalhas-Almeida C Serra J Moita J Cavadas C Alvaro AR. Understanding neuron-glia crosstalk and biological clocks in insomnia. Neurosci Biobehav Rev. (2023) 147:105100. doi: 10.1016/j.neubiorev.2023.105100, 36804265

[4] Rivero-Segura NA Cuartas JDR Garcia-delaTorre P Sanchez-Garcia S Ramirez-Aldana R Gomez-Verjan JC. Insomnia accelerates the epigenetic clocks in older adults. Geroscience. (2025) 47:6777–88. doi: 10.1007/s11357-025-01608-7, 40100530 PMC12638505

[5] Schutte-Rodin S Broch L Buysse D Dorsey C Sateia M. Clinical guideline for the evaluation and Management of Chronic Insomnia in adults. J Clin Sleep Med. (2008) 4:487–504. Epub 2008/10/16. doi: 10.5664/jcsm.27286, 18853708 PMC2576317

[6] Ye Z Liang M Hu Q Yu Y Wang S Qiu H. Research progress of insomnia disorder at home and abroad. Medic Philosophy (B). (2017) 38:60–3.

[7] Vincent N Lionberg C. Treatment preference and patient satisfaction in chronic insomnia. Sleep. (2001) 24:411–7. doi: 10.1093/sleep/24.4.411, 11403525

[8] de Jong M Spinhoven P Korrelboom K Deen M van der Meer I Danner UN . Effectiveness of enhanced cognitive behavior therapy for eating disorders: a randomized controlled trial. Int J Eat Disord. (2020) 53:447–57. doi: 10.1002/eat.23239, 32040244 PMC7317943

[9] Kooistra LC Wiersma JE Ruwaard J Neijenhuijs K Lokkerbol J van Oppen P . Cost and effectiveness of blended versus standard cognitive Behavioral therapy for outpatients with depression in routine specialized mental health care: pilot randomized controlled trial. J Med Internet Res. (2019) 21:e14261. doi: 10.2196/14261, 31663855 PMC6914243

[10] Soong C Burry L Greco M Tannenbaum C. Advise non-pharmacological therapy as first line treatment for chronic insomnia. BMJ. (2021) 372:n680. doi: 10.1136/bmj.n680, 33757960

[11] Yeung WF Yu BY Yuen JW Ho JYS Chung KF Zhang ZJ . Semi-individualized acupuncture for insomnia disorder and oxidative stress: a randomized, double-blind, sham-controlled trial. Nat Sci Sleep. (2021) 13:1195–207. doi: 10.2147/nss.S318874, 34321944 PMC8310926

[12] Wang C Xu WL Li GW Fu C Li JJ Wang J . Impact of acupuncture on sleep and comorbid symptoms for chronic insomnia: a randomized clinical trial. Nat Sci Sleep. (2021) 13:1807–22. doi: 10.2147/nss.S326762, 34675728 PMC8519353

[13] Yu Y Li X Zhu Z Wang Y Xi Q Qiu J . Acupuncture for chronic insomnia disorder: a systematic review with Meta-analysis and trial sequential analysis. Front Neurol. (2025) 16:1541276. doi: 10.3389/fneur.2025.1541276, 40371085 PMC12074954

[14] Kim S-A Lee S-H Kim J-H Van Den Noort M Bosch P Won T . Efficacy of acupuncture for insomnia: a systematic review and meta-analysis. Am J Chin Med. (2021) 49:1135–50. doi: 10.1142/S0192415X21500543, 34049475

[15] Liu C Zhao Y Qin S Wang X Jiang Y Wu W. Randomized controlled trial of acupuncture for anxiety and depression in patients with chronic insomnia. Ann Transl Med. (2021) 9:1426. doi: 10.21037/atm-21-3845, 34733978 PMC8506741

[16] Wang YK Li T Ha LJ Lv ZW Wang FC Wang ZH . Effectiveness and cerebral responses of multi-points acupuncture for primary insomnia: a preliminary randomized clinical trial and Fmri study. BMC Complement Med Ther. (2020) 20:254. doi: 10.1186/s12906-020-02969-6, 32807158 PMC7430003

[17] Mai E Buysse DJ. Insomnia: prevalence, impact, pathogenesis, differential diagnosis, and evaluation. Sleep Med Clin. (2008) 3:167–74. doi: 10.1016/j.jsmc.2008.02.001, 19122760 PMC2504337

[18] Haest K Kumar A Van Calster B Leunen K Smeets A Amant F . Stellate ganglion block for the management of hot flashes and sleep disturbances in breast cancer survivors: an uncontrolled experimental study with 24 weeks of follow-up. Ann Oncol. (2012) 23:1449–54. doi: 10.1093/annonc/mdr478, 22039079

[19] Hanling SR Hickey A Lesnik I Hackworth RJ Stedje-Larsen E Drastal CA . Stellate ganglion block for the treatment of posttraumatic stress disorder: a randomized, double-blind, controlled trial. Reg Anesth Pain Med. (2016) 41:494–500. doi: 10.1097/aap.0000000000000402, 27187898

[20] Ren Y Zhang Z Li HP Zhang PJ Duo J Kong H. A comprehensive overview of the stellate ganglion block throughout the past three decades: a bibliometric analysis. Pain Physician. (2024) 27:E597-e610 39087973

[21] Y XQ Z WT H J L H. Musculoskeletal ultrasound-guided acupuncture of the stellate ganglion for refractory insomnia: a case report. Chin J Rural Med Pharm. (2024) 31:14–5. doi: 10.19542/j.cnki.1006-5180.2312-479

[22] Cui X Bray S Reiss AL. Functional near infrared spectroscopy (Nirs) signal improvement based on negative correlation between oxygenated and deoxygenated Hemoglobin dynamics. NeuroImage. (2010) 49:3039–46. doi: 10.1016/j.neuroimage.2009.11.050, 19945536 PMC2818571

[23] Altena E Van Der Werf YD Sanz-Arigita EJ Voorn TA Rombouts SA Kuijer JP . Prefrontal hypoactivation and recovery in insomnia. Sleep. (2008) 31:1271–6. 18788652 PMC2542967

[24] Nofzinger EA Buysse DJ Germain A Price JC Miewald JM Kupfer DJ. Functional neuroimaging evidence for hyperarousal in insomnia. Am J Psychiatry. (2004) 161:2126–8. doi: 10.1176/appi.ajp.161.11.2126, 15514418

[25] Gong H Sun H Ma Y Tan Y Cui M Luo M . Prefrontal brain function in patients with chronic insomnia disorder: a pilot functional near-infrared spectroscopy study. Front Neurol. (2022) 13:985988. doi: 10.3389/fneur.2022.985988, 36588900 PMC9798108

[26] Zhou Q Li C Yu J Tang Y Liu Z Qi G . Cortical activation and functional connectivity during a verbal fluency task in patients with chronic insomnia: a Multi-Channel Nirs study. J Psychiatr Res. (2024) 179:270–8. doi: 10.1016/j.jpsychires.2024.09.025, 39332354

[27] Sun J-J Liu X-M Shen C-Y Zhang X-Q Sun G-X Feng K . Reduced prefrontal activation during verbal fluency task in chronic insomnia disorder: a multichannel near-infrared spectroscopy study. Neuropsychiatr Dis Treat. (2017) 13:1723–31. doi: 10.2147/NDT.S136774, 28721053 PMC5501642

[28] Shao Z Janse E Visser K Meyer AS. What do verbal fluency tasks measure? Predictors of verbal fluency performance in older adults. Front Psychol. (2014) 5:1–10. doi: 10.3389/fpsyg.2014.00772 25101034 PMC4106453

[29] Ren Y Cui C Feng K Zhang X Yu C Liu P. A scoping review of utilization of the verbal fluency task in Chinese and Japanese clinical settings with near-infrared spectroscopy. Front Psychiatry. (2024) 15:1–15. doi: 10.3389/fpsyt.2024.1282546 PMC10957746 38525251

[30] Schulz KF Altman DG Moher D. Consort 2010 statement: updated guidelines for reporting parallel group randomized trials. Ann Intern Med. (2010) 152:726–32. doi: 10.7326/0003-4819-152-11-201006010-00232, 20335313

[31] MacPherson H Altman DG Hammerschlag R Youping L Taixiang W White A . Revised standards for reporting interventions in clinical trials of acupuncture (Stricta): extending the Consort statement. PLoS Med. (2010) 7:e1000261. doi: 10.1371/journal.pmed.1000261, 21349059

[32] Yang M Morin CM Schaefer K Wallenstein GV. Interpreting score differences in the insomnia severity index: using health-related outcomes to define the minimally important difference. Curr Med Res Opin. (2009) 25:2487–94. doi: 10.1185/03007990903167415, 19689221

[33] Yin X Gou M Xu J Dong B Yin P Masquelin F . Efficacy and safety of acupuncture treatment on primary insomnia: a randomized controlled trial. Sleep Med. (2017) 37:193–200. doi: 10.1016/j.sleep.2017.02.012, 28899535

[34] Tamai H Komine S Kikuchi S Waki H. Exploring feasibility of Fnirs to assess delayed inhibition effect of prefrontal cortex for acute stress by acupuncture on Gv20: a pilot study. Front Hum Neurosci. (2024) 18:1433312. doi: 10.3389/fnhum.2024.1433312, 39619678 PMC11604711

[35] Si X Xiang S Zhang L Li S Zhang K Ming D. Acupuncture with Deqi modulates the hemodynamic response and functional connectivity of the prefrontal-motor cortical network. Front Neurosci. (2021) 15:693623. doi: 10.3389/fnins.2021.693623, 34483822 PMC8415569

[36] Fernandez Rojas R Liao M Romero J Huang X Ou KL. Cortical network response to acupuncture and the effect of the Hegu point: an Fnirs study. Sensors (Basel). (2019) 19:1–16. doi: 10.3390/s19020394, 30669377 PMC6359459

[37] Zhou W Benharash P. Significance of "Deqi" response in acupuncture treatment: myth or reality. J Acupunct Meridian Stud. (2014) 7:186–9. doi: 10.1016/j.jams.2014.02.008, 25151451

[38] Hui KK Nixon EE Vangel MG Liu J Marina O Napadow V . Characterization of the" Deqi" response in acupuncture. BMC Complement Altern Med. (2007) 7:33. doi: 10.1186/1472-6882-7-33, 17973984 PMC2200650

[39] Yuan HW Ma LX Zhang P Lin C Qi DD Li J . An exploratory survey of Deqi sensation from the views and experiences of Chinese patients and acupuncturists. Evid Based Complement Alternat Med. (2013) 2013:430851. doi: 10.1155/2013/430851, 24348700 PMC3857737

[40] Gofeld M Bhatia A Abbas S Ganapathy S Johnson M. Development and validation of a new technique for ultrasound-guided stellate ganglion block. Reg Anesth Pain Med. (2009) 34:475–9. doi: 10.1097/AAP.0b013e3181b494de, 19920422

[41] Bhatia A Flamer D Peng PW. Evaluation of sonoanatomy relevant to performing stellate ganglion blocks using anterior and lateral simulated approaches: an observational study. Can J Anesth/J Can Anesth. (2012) 59:1040–7. doi: 10.1007/s12630-012-9779-4, 22956268

[42] Shan H-H Chen H-F Ni Y Yang J-X Zhou X-L. Effects of stellate ganglion block through different approaches under guidance of ultrasound. Front Surg. (2022) 8:797793. doi: 10.3389/fsurg.2021.797793, 35111806 PMC8801483

[43] Langevin HM Konofagou EE Badger GJ Churchill DL Fox JR Ophir J . Tissue displacements during acupuncture using ultrasound elastography techniques. Ultrasound Med Biol. (2004) 30:1173–83. doi: 10.1016/j.ultrasmedbio.2004.07.010, 15550321

[44] Piper SK Krueger A Koch SP Mehnert J Habermehl C Steinbrink J . A wearable multi-channel Fnirs system for brain imaging in freely moving subjects. NeuroImage. (2014) 85 Pt) 85 Pt 1:64–71. doi: 10.1016/j.neuroimage.2013.06.062, 23810973 PMC3859838

[45] Strangman G Culver JP Thompson JH Boas DA. A quantitative comparison of simultaneous bold Fmri and Nirs recordings during functional brain activation. NeuroImage. (2002) 17:719–31. Epub 2002/10/16. doi: 10.1006/nimg.2002.1227, 12377147

[46] Fortier-Brochu E Beaulieu-Bonneau S Ivers H Morin CM. Insomnia and daytime cognitive performance: a meta-analysis. Sleep Med Rev. (2012) 16:83–94. doi: 10.1016/j.smrv.2011.03.008, 21636297

[47] Johnson DA Knutson K Colangelo LA Hale L Redline S Carnethon M . Associations of chronic burden, sleep characteristics, and metabolic syndrome in the coronary artery risk development in young adults study. Psychosom Med. (2022) 84:711–8. doi: 10.1097/psy.0000000000001081, 35420593 PMC9271585

[48] Meng L Zheng Y Hui R. The relationship of sleep duration and insomnia to risk of hypertension incidence: a meta-analysis of prospective cohort studies. Hypertens Res. (2013) 36:985–95. doi: 10.1038/hr.2013.70, 24005775 PMC3819519

[49] Vgontzas AN Liao D Pejovic S Calhoun S Karataraki M Bixler EO. Insomnia with objective short sleep duration is associated with type 2 diabetes: a population-based study. Diabetes Care. (2009) 32:1980–5. doi: 10.2337/dc09-0284, 19641160 PMC2768214

[50] Freeman D Sheaves B Waite F Harvey AG Harrison PJ. Sleep disturbance and psychiatric disorders. Lancet Psychiatry. (2020) 7:628–37. doi: 10.1016/s2215-0366(20)30136-x, 32563308

[51] Liu M Hou T Nkimbeng M Li Y Taylor JL Sun X . Associations between symptoms of pain, insomnia and depression, and frailty in older adults: a cross-sectional analysis of a cohort study. Int J Nurs Stud. (2021) 117:103873. doi: 10.1016/j.ijnurstu.2021.103873, 33621722 PMC9940903

[52] Garbarino S Lanteri P Bragazzi NL Magnavita N Scoditti E. Role of sleep deprivation in immune-related disease risk and outcomes. Commun Biol. (2021) 4:1304. doi: 10.1038/s42003-021-02825-4, 34795404 PMC8602722

[53] Perlis ML Vargas I Ellis JG Grandner MA Morales KH Gencarelli A . The natural history of insomnia: the incidence of acute insomnia and subsequent progression to chronic insomnia or recovery in good sleeper subjects. Sleep. (2020) 43:1–16. doi: 10.1093/sleep/zsz299, 31848629 PMC7294401

[54] Taylor DJ Mallory LJ Lichstein KL Durrence HH Riedel BW Bush AJ. Comorbidity of chronic insomnia with medical problems. Sleep. (2007) 30:213–8. doi: 10.1093/sleep/30.2.213, 17326547

[55] Yeung W-F Chung K-F Poon MM-K Ho FY-Y Zhang S-P Zhang Z-J . Prescription of Chinese herbal medicine and selection of acupoints in pattern-based traditional Chinese medicine treatment for insomnia: a systematic review. Evid Based Complement Alternat Med. (2012) 2012:902578. doi: 10.1155/2012/902578, 23259001 PMC3521464

[56] Cao H-J Yu M-L Wang L-Q Fei Y-T Xu H Liu J-P. Acupuncture for primary insomnia: an updated systematic review of randomized controlled trials. J Altern Complement Med. (2019) 25:451–74. doi: 10.1089/acm.2018.0046, 31013432

[57] Song X-J Zhu Y-H Wu P Du L Li Z-W. Acupoint compatibility effect and mechanism of Shenmen (Ht7) and Sanyinjiao (Sp6) in improving daytime fatigue and sleepiness of insomnia. Zhen Ci Yan Jiu. (2022) 47:630–5. doi: 10.13702/j.1000-0607.20210590, 35880281

[58] Yao L Liu Y Li M Zheng H Sun M He M . The central regulatory effects of acupuncture in treating primary insomnia: a review. Front Neurol. (2024) 15:1406485. doi: 10.3389/fneur.2024.1406485, 39719980 PMC11666528

[59] Yan S Wang Y Yu L Xia W Xue F Yu Y . Stellate ganglion block alleviates postoperative sleep disturbance in patients undergoing radical surgery for gastrointestinal malignancies. J Clin Sleep Med. (2023) 19:1633–42. doi: 10.5664/jcsm.10632, 37128727 PMC10476041

[60] Takamiya T Kubo Y Benharash P Zhou W. Effect of electroacupuncture on porcine cardiac excitability induced by left stellate ganglion stimulation. Auton Neurosci. (2018) 213:15–22. doi: 10.1016/j.autneu.2018.05.005, 30005736

[61] Uchida S Kagitani F Hotta H. Neural mechanisms of reflex inhibition of heart rate elicited by acupuncture-like stimulation in anesthetized rats. Auton Neurosci. (2010) 157:18–23. doi: 10.1016/j.autneu.2010.03.021, 20460195

[62] Lee S Kim SN. The effects of acupuncture on sleep disorders and its underlying mechanism: a literature review of rodent studies. Front Neurosci. (2023) 17:1243029. doi: 10.3389/fnins.2023.1243029, 37614343 PMC10442542

